# FedLC-Trans: privacy-preserving CNN–Transformer fusion for resilient IoMT intrusion detection

**DOI:** 10.3389/fpubh.2026.1924540

**Published:** 2026-09-25

**Authors:** Zhi Gong, Naixue Xiong, Chenglin Zhao, Jiao Chen, Oad Ammar, Jamshed Ali Shaikh, Reem Ibrahim Alkanhel, Mahmood Al-Bahri, Mohammed Muthanna

**Affiliations:** 1 School of Computer Science and Engineering, Hunan University of Science and Technology, Xiangtan, China; 2 Key Laboratory of Data and Model Security of Hunan Province for Higher Education Institutions (Hunan University of Information Technology), Changsha, China; 3 Department of Computer Science, Southern New Hampshire University, Manchester, NH, United States; 4 School of Computer Science and Engineering, Hunan University of Information Technology, Changsha, China; 5 School of Information Science and Engineering, Shaoyang University, Shaoyang, Hunan, China; 6 School of Computer Science and Engineering, South China University of Technology, Guangzhou, Guangdong, China; 7 Department of Information Technology, College of Computer and Information Sciences, Princess Nourah Bint Abdulrahman University, Riyadh, Saudi Arabia; 8 Faculty of Computing and IT, Sohar University, Sohar, Oman

**Keywords:** CNN, cybersecurity, federated learning, IoMT, Transformer

## Abstract

The objective of this study is to develop an accurate and computationally efficient intrusion detection framework for distributed Internet of Medical Things (IoMT) environments while reducing the need to centralize raw client data. We propose FedLC-Trans, which integrates MobileNet-based lightweight convolutional blocks for local feature extraction, an Efficient Transformer for modeling interactions and dependencies among feature-token representations, and an attention-guided SeqPool module for discriminative feature aggregation. Federated Averaging (FedAvg) is employed to collaboratively train the model across five non-IID clients without transferring raw client data to the central server. To reduce the risk of evaluation leakage, normalization, Random Forest-based feature selection, PCA, and SMOTE are derived or applied using the training data only, while the learned transformations are subsequently applied to the held-out test data. FedLC-Trans was evaluated on the WUSTL-EHMS, ECU-IoHT, and CICIoMT2024 datasets and achieved accuracies of 0.9913, 0.9892, and 0.9916, respectively. The results indicate consistently high intrusion-detection performance across the three evaluated IoMT datasets. However, computational profiling was performed on an NVIDIA A100 GPU rather than on resource-constrained IoMT edge hardware, and federated learning alone does not provide formal protection against gradient leakage, model inversion, or membership-inference attacks. Therefore, the present findings demonstrate reduced centralized raw-data exposure and promising computational characteristics, while edge-device validation and stronger privacy-preserving mechanisms remain important directions for future work.

## 1 Introduction

The rapid growth of connected devices has accelerated the development of Internet of Things (IoT)-enabled services across many sectors. The number of smart devices with internet connectivity is projected to reach 27 billion by 2027, reflecting an annual growth rate of approximately 14% (1). Similarly, the global number of connected IoT devices is expected to reach 30 billion by 2026, more than double the number reported in 2021 (2). In healthcare, this growth has contributed to the emergence of the Internet of Medical Things (IoMT), where medical devices, sensors, and healthcare applications are interconnected to support remote monitoring, diagnosis, and treatment (3). By enabling continuous collection, transmission, and processing of biomedical and network data, IoMT has become an important component of modern healthcare delivery (4, 5).

IoMT systems improve healthcare services by supporting real-time monitoring, efficient data management, personalized care, and reduced operational costs (6, 7). However, deep learning-based IoMT systems are vulnerable to cyberattacks that may compromise sensitive patient data and disrupt critical healthcare services (8). Therefore, robust security mechanisms are essential to protect patient-centered IoMT environments. Machine learning (ML) methods, including support vector machines and random forests, have been widely used for IDS tasks because of their scalability and classification ability (9). However, these approaches often depend on handcrafted features and may struggle to represent complex traffic patterns in dynamic IoMT environments (10, 11). Deep learning (DL) methods provide stronger feature learning capability and have been increasingly applied to IoMT intrusion detection (12–15). Convolutional neural networks (CNNs) are effective for extracting local spatial features, while recurrent and attention-based models can capture sequential relationships in traffic data (16, 17). Nevertheless, existing models often face a trade-off between detection performance and computational cost, which limits their deployment on resource-constrained IoMT devices.

Federated learning (FL) has emerged as a promising approach for privacy-aware learning in distributed IoT and IoMT environments. Instead of collecting raw patient or traffic data at a centralized server, FL enables clients to train models locally and share only model parameters for aggregation. This reduces the privacy risks associated with centralized data collection while allowing collaborative learning across distributed devices. However, FL-based IDS still faces practical challenges, including model synchronization, communication overhead, heterogeneous client data, and scalability in real-time healthcare networks.

Several recent IDS studies have explored CNN, Transformer, and FL-based approaches for IoMT security. However, many methods prioritize either detection accuracy or model efficiency, while fewer works jointly address lightweight spatial–temporal feature learning and decentralized training. To address this gap, we propose FedLC-Trans, a federated intrusion detection framework that integrates a lightweight LC-Trans backbone with federated learning. LC-Trans combines MobileNet-based CNN layers for spatial feature extraction, Efficient Transformer blocks for temporal dependency modeling, and a SeqPool module for attention-guided feature compression. The federated training strategy enables distributed IoMT clients to collaborate without transferring raw data to a central server.

Figure 1 illustrates the comparative evolution of intrusion detection system (IDS) architectures within IoMT healthcare environments. Figure 1a represents the traditional, standalone IDS workflow that passes centralized data through basic pre-processing and standalone machine/deep learning classifiers. Figure 1b shows the existing centralized IoMT-IDS workflow, where distributed edge and gateway medical devices are forced to transmit sensitive patient data to a cloud-based healthcare system for anomaly detection. In contrast, Figure 1c presents the proposed decentralized FedLC-Trans framework, which preserves data privacy by keeping datasets local, extracting spatial-temporal features via an integrated MobileNet CNN, Efficient Transformer, and SeqPool pipeline (Stages 1 and 2), and securely aggregating model updates in the cloud (Stage 3) to execute robust, privacy-preserving intrusion detection (Stage 4).

**Figure 1:**
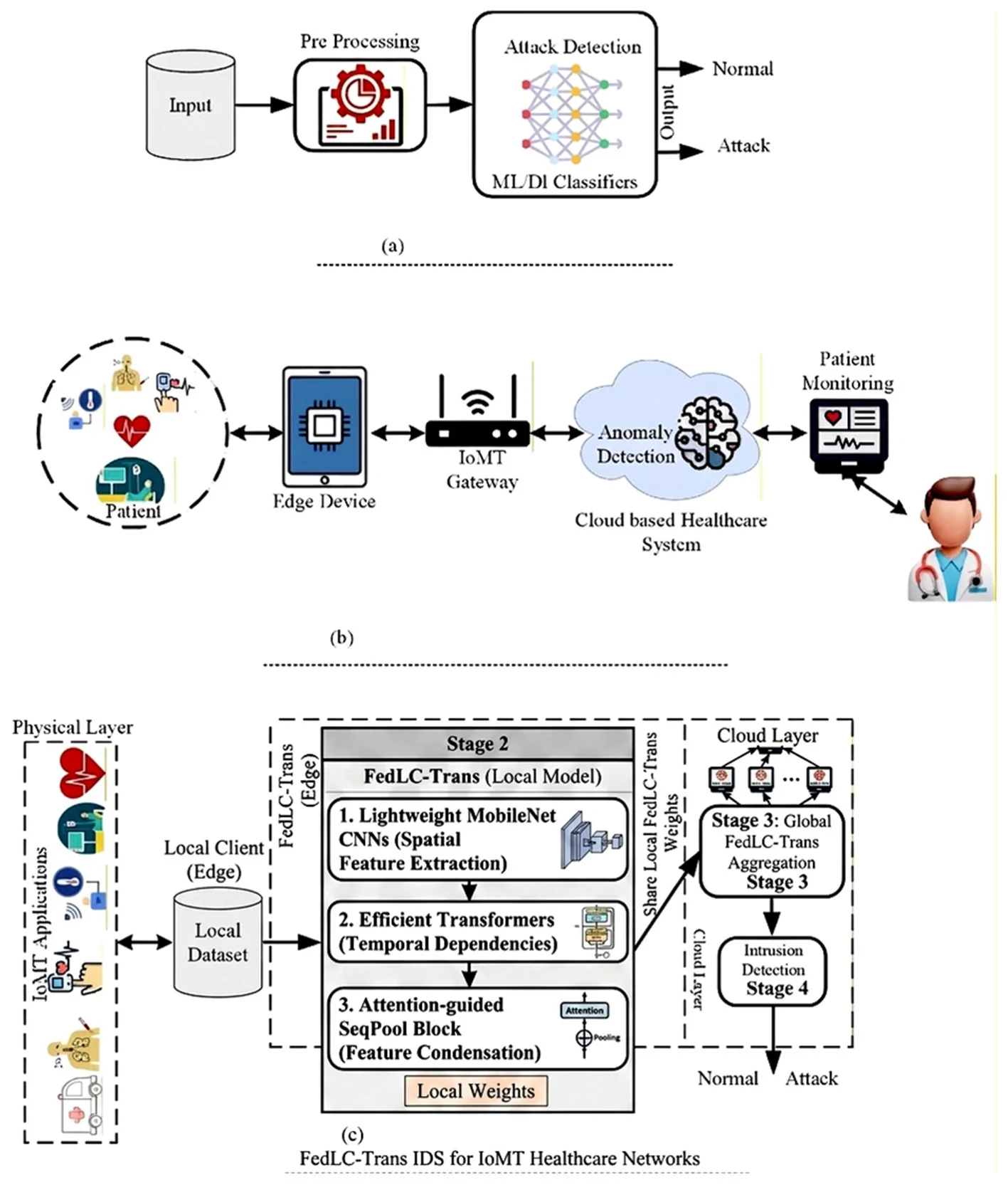
Existing IDS models for IoMT. **(a)** Existing IDS workflow. **(b)** Existing centralized IDS workflow. **(c)** Proposed federated learning-based FedLC-Trans for IoMT healthcare networks.

The key contributions of this study can be summarized as follows.

- We designed FedLC-Trans, a novel module that combines Lightweight Convolutional Neural Networks (CNN) and Efficient Transformers for IoMT-based IDS. This hybrid architecture is specifically designed to efficiently detect cyberattacks in IoMT healthcare networks, ensuring robust, situationally adaptive monitoring within IoMT healthcare networks. In real-time scenarios, this framework can monitor and protect IoMT systems continuously, providing immediate detection of cyber threats in dynamic healthcare environments.
- We introduce LC-Trans, which incorporates Lightweight MobileNet CNNs for early-stage spatial feature extraction, identifying low-level attack patterns such as anomalies in network traffic and device logs. In subsequent stages, Efficient Transformers capture temporal dependencies to detect complex, time-based attack patterns that unfold over multiple events. The obtained high-level features are processed through an attention-guided SeqPool block, which compresses the features and applies attention mechanisms. This mechanism efficiently selects and compresses the most discriminative features, optimizing the model's ability to focus on key patterns while minimizing computational complexity.
- We integrate federated learning into the proposed IDS framework to enable collaborative model training across distributed IoMT clients. Instead of transferring raw client data to a central server, each client trains locally and shares model weight updates for FedAvg aggregation. This reduces centralized raw-data exposure while supporting distributed intrusion detection in healthcare-oriented IoMT environments.
- We evaluate FedLC-Trans on three publicly available IoMT datasets and demonstrate strong detection performance across multiple attack categories while maintaining computational efficiency.

The paper includes: Section 2 on related work, Section 3 on FedLC-Trans, Section 4 on Experimental analysis, Section 5 on Results, and Section 6 on Conclusions.

## 2 Related work

The rapid growth of IoMT has increased the need for reliable intrusion detection systems in healthcare networks. IoMT environments generate heterogeneous traffic from medical sensors, wearable devices, gateways, and cloud services, creating new cybersecurity risks for patient data and clinical operations. Therefore, IDS methods for IoMT must balance detection accuracy, computational efficiency, scalability, and privacy preservation. Existing IDS approaches for IoMT can be broadly grouped into three categories: centralized machine learning (ML), deep learning (DL), and federated learning (FL). Centralized ML methods, such as support vector machines, random forests, and XGBoost, are commonly used for traffic classification because they are relatively simple and interpretable. However, these methods often depend on handcrafted features and may struggle to capture complex spatial and temporal traffic patterns. DL-based methods, including CNN (18), LSTM, CNN-LSTM, and Transformer models, improve feature learning capability and can model more complex attack patterns. Nevertheless, many DL models require centralized training and high computational resources, which limits their deployment in resource-constrained IoMT environments.

As summarized in Table 1, recent IDS studies have reported strong performance on different IoT and IoMT datasets.

**Table 1:** Summary of representative IDS methodologies for IoT/IoMT security.

No.	Method	Dataset	Benefits	Limitations	Reported Acc
1	CNN (40)	ECU-IoHT	Optimized hyperparams	High compute	0.9885
2	Meta-IDS (41)	IoMT	High detection	Complex design	0.9991
3	CNN+LSTM (42)	ToN-IoT	Multi-class acc	Overfitting	0.9998
4	ML models (43)	CICIoT/CICIoMT	Temporal dep.	Dataset challenges	0.9985
5	IDS (44)	WUSTL/ECU-IoHT	Strong security	Scalability	0.9901
6	Transformer (45)	WUSTL	High perf	Privacy limits	0.9913
7	CNN-Transformer (46)	WUSTL-EHMS	Robust detection	Complex data	0.9998
8	Transformer (47)	WUSTL-EHMS	Reliable det.	High compute	0.9994
9	DL models (48)	IoMT	Imbalance handling	Low large-dataset acc	0.9980
10	FL (26)	CICIoMT	Dynamic averaging	Limited temporal modeling	0.9922
11	XGBoost (19)	WUSTL-EHMS	Interpretability	Implementation	0.9853
12	CNN-LSTM+RL (20)	CICIoMT	Adaptive temporal	High compute	0.9958
13	MF-Transformer (21)	ECU/WUSTL-EHMS	Long-range dep.	Complex	0.9988
14	CNN-Transformer Attention (22)	ECU/WUSTL-EHMS	Effective anomaly	Imbalance issues	0.9978
15	FL (23)	ToN-IoT	Scalable	Limited IoMT relevance	0.9931
16	Meta-Fed IDS (24)	Custom WUSTL-EHMS	Meta-learning	Few clients	0.9912
17	FederatedAve (25)	NF-UNSW/NF-BoT-IoT	Collaboration	Not IoMT-specific	0.9461

Several studies have focused on centralized IDS models for IoMT security. FOID (19) applies recursive feature elimination to improve feature selection and detection performance. HCLR-IDS (20) combines CNN-based learning with reinforcement learning for adaptive threat response. Transformer-based models, such as MF-Transformer (21), use attention mechanisms to capture long-range dependencies in traffic data, while RCLNet (22) combines Random Forest-based feature selection with CNN-attention mechanisms to address traffic imbalance. Although these models show strong classification performance, many of them remain computationally demanding and rely on centralized data collection, which raises privacy concerns in healthcare settings.

Federated learning has been introduced to address privacy concerns by allowing distributed clients to train models locally without sharing raw data. Singh et al. (23) proposed a Dew-Cloud-based hierarchical FL framework for IoMT security, distributing computation across dew servers, fog nodes, and cloud servers. Although the framework demonstrated scalability, its reliance on older datasets may limit its relevance to modern IoMT traffic patterns. Meta-Fed IDS (24) introduced a meta-learning-based FL framework using decision tree, AdaBoost, random forest, and XGBoost components, but its evaluation was limited to three clients and did not fully address non-IID data conditions. Similarly, Sarhan et al. (25) developed a federated threat intelligence framework using FedAvg with LSTM and DNN models, but the method was designed mainly for general IoT contexts rather than IoMT-specific heterogeneity.

Other recent FL-based IDS approaches have attempted to improve communication efficiency and resource awareness. Misbah et al. (26) proposed a dynamic averaging strategy using random forest classifiers for medical IoT, but RF-based models have limited ability to capture temporal dependencies. Ioannou et al. (27) introduced GEMLIDS-MIoT, an energy-efficient FL framework based on a pruned enhanced random forest and one-class SVM for deployment on Raspberry Pi gateways. Khan et al. (28) proposed FedInforce-Fusion, which integrates reinforcement learning to reduce communication overhead. These studies show the potential of FL for privacy-aware IDS, but challenges remain in balancing detection performance, computational efficiency, communication cost, and heterogeneous client data.

Overall, the reviewed literature shows that centralized DL models can achieve strong detection performance, while FL-based frameworks provide stronger privacy support for distributed IoMT environments. However, many existing methods still face limitations related to computational overhead, non-IID client distributions, IoMT-specific scalability, and efficient spatial-temporal feature learning. These gaps motivate the proposed FedLC-Trans framework, which combines a lightweight LC-Trans backbone with federated learning to support efficient and privacy-aware intrusion detection in IoMT networks.

## 3 Proposed FedLC-Trans

This section presents the proposed FedLC-Trans framework for intrusion detection in IoMT networks. First, the overall system workflow is introduced, including leakage-free preprocessing and distributed model training. Next, the FedLC-Trans architecture is described in detail, including the MobileNet-based convolutional block, the Efficient Transformer block, the SeqPool feature-compression module, and the final classification layer. Finally, the federated learning procedure is explained, including local client training, FedAvg aggregation, and global model inference.

### 3.1 System overview

The proposed FedLC-Trans framework in Figure 2 provides a privacy-preserving, efficient intrusion detection system for IoMT healthcare networks. IoMT traffic data, which first undergoes a leakage-free preprocessing pipeline including SMOTE-based class balancing, feature normalization, Random Forest feature selection, and PCA dimensionality reduction to improve data quality and reduce computational complexity (Stage 1). In Stage 2, each client trains a local LC-Trans model that combines Lightweight CNNs for spatial feature extraction with Efficient Transformers for temporal pattern learning. Only the model updates, not raw data, are shared with the cloud for aggregation (Stage 3), producing a global model. Stage 4 applies the aggregated model to classify traffic as normal or attack. Fog nodes deploy the trained models locally to detect intrusions in real time, forwarding alerts to the cloud for coordination. By integrating federated learning with spatial-temporal feature modeling and attention-based compression, FedLC-Trans achieves high detection accuracy while preserving data privacy and supporting collaborative monitoring across distributed IoMT environments.

**Figure 2:**
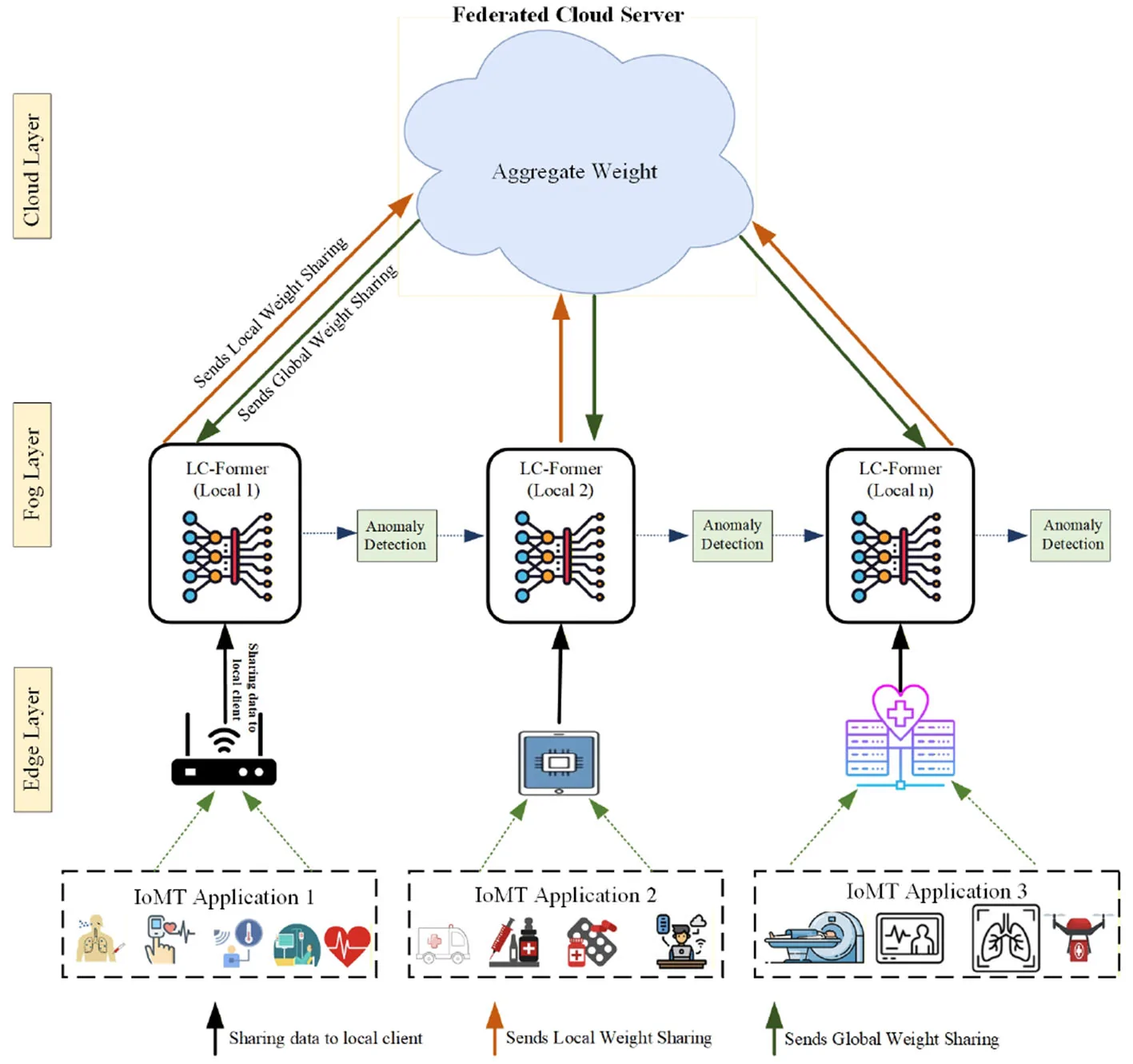
Overall proposed architecture.

### 3.2 Data preprocessing

To address the risk of data leakage, all preprocessing operations are performed after the train-test split. Each dataset is first divided into 70% training and 30% testing subsets. Normalization parameters are fitted only on the training set and then applied to the testing set. SMOTE is applied only to the training set to balance minority classes; no synthetic samples are generated from testing data. Random Forest-based feature importance is also computed only from the training set, and the selected feature indices are then applied to both training and testing data. Similarly, PCA is fitted only on the training set and subsequently used to transform the testing set. This workflow ensures that no information from the testing set is used during balancing, scaling, feature selection, or dimensionality reduction summarized in Algorithm 1.

Algorithm 1 **Leakage-Free Preprocessing**

 **Require:**  Raw dataset *D*
 **Ensure:**  Preprocessed training set *D*_train_ and testing set *D*_test_
 1:  Split *D* into *D*_train_ (70%) and *D*_test_ (30%).
 2:  Fit normalization parameters on *D*_train_ only.
 3:  Transform *D*_train_ and *D*_test_ using training-fitted normalization parameters.
 4:  Apply SMOTE only to *D*_train_.
 5:  Fit the Random Forest feature selector on *D*_train_ only.
 6:  Apply the selected feature indices to *D*_train_ and *D*_test_.
 7:  Fit PCA on *D*_train_ only.
 8:  Transform *D*_train_ and *D*_test_ using the PCA model fitted on *D*_train_.
 9:  **return** *D*_train_, *D*_test_.

For the federated learning experiments, feature-space consistency across clients was explicitly maintained before FedAvg aggregation. Specifically, normalization, Random Forest-based feature selection, and PCA were fitted only on the global training partition and never on the testing partition. The selected feature indices, PCA projection matrix, and normalization parameters learned from the training partition were then applied consistently to all client training subsets and to the held-out testing set. After this common training-derived feature space was established, the transformed training samples were partitioned into five non-IID client subsets. SMOTE was applied only within each client's local training subset to address class imbalance; no synthetic samples were generated from testing data. This procedure prevents test-set leakage while ensuring that all clients share the same input dimensionality and feature representation during FedAvg aggregation.

### 3.3 LC-Trans

LC-Trans is the lightweight CNN-Transformer backbone used in the proposed FedLC-Trans framework. It is designed to extract compact spatial-temporal representations from IoMT traffic while maintaining low computational cost for resource-constrained healthcare environments. As illustrated in Figure 3, LC-Trans follows a unified architecture composed of a MobileNet-based convolutional block, an Efficient Transformer block, a SeqPool feature-compression module.

**Figure 3:**
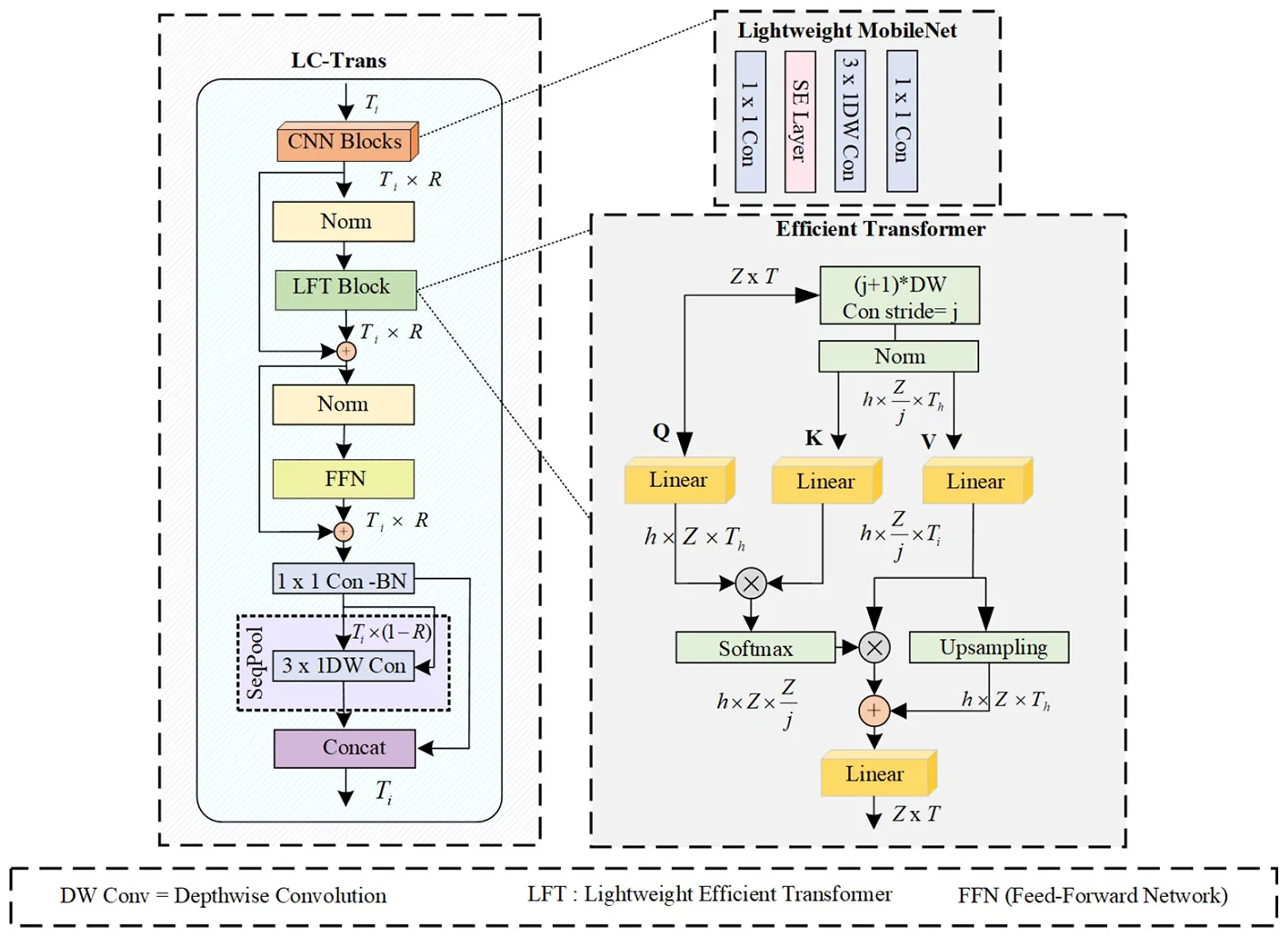
Proposed LC-Trans encoder architecture. DW Conv, depthwise convolution; LFT, lightweight efficient transformer; FFN, feed-forward network.

Recent lightweight CNN architectures, including MobileNet (29), EfficientNetV2 (30, 31), ShuffleNetV2 (32), and ResNet (33), have shown that efficient network design can reduce parameter count and computational overhead while preserving feature extraction ability. Among them, MobileNet is particularly suitable for IoMT environments because its depthwise separable convolutions substantially reduce computation compared with conventional convolutions. However, CNN-based models mainly capture local spatial patterns and may not fully model longer-range dependencies in traffic sequences. Transformer-based models are effective for capturing global and temporal relationships through self-attention (21), but standard Transformers are computationally expensive and difficult to deploy on resource-limited IoMT devices.

The MobileNet-based convolutional block first extracts local patterns from the processed IoMT feature representation using depthwise separable convolutions, linear bottlenecks, and Squeeze-and-Excitation (SE) channel recalibration (34). The resulting representation is subsequently organized into feature tokens and passed to the Efficient Transformer block. Importantly, these tokens do not represent consecutive timestamps, packets, flows, or other chronologically ordered network events. Instead, they correspond to positions or groups within the processed feature representation. Consequently, the self-attention mechanism is employed to model interactions and dependencies among feature-token representations rather than temporal dependencies among successive network events. This distinction is important because reshaping or tokenizing a fixed-dimensional feature vector does not inherently introduce temporal information. The Efficient Transformer therefore captures relationships among the learned feature tokens while reducing the computational complexity associated with the attention mechanism. Finally, the resulting token-level representations are processed by the SeqPool module, which assigns learned importance weights to the tokens and aggregates them into a compact discriminative representation for final intrusion classification. Input feature vectors of dimension *D*_raw_ = 120 are reshaped into sequences of length *L* = 30 for processing by the Efficient Transformer block. After PCA or feature selection, the final feature dimension is reduced to *D* = 64. Each token in the sequence corresponds to a segment of network traffic features over a short time interval, ensuring that the Transformer can model temporal dependencies in IoMT traffic effectively.

The LC-Trans backbone begins with a 3 × 1 convolution with stride 2 to generate low-level feature representations, followed by a second 3 × 1 convolution with stride 1 to refine local patterns. An overlapping patch-embedding layer is then applied using a 3 × 1 convolution with stride 2 and layer normalization. This operation reduces sequence length while increasing the channel dimension, producing a compact representation for Transformer processing.

Let the input sequence to the Efficient Transformer block be *X* ∈ ℝ^*L*×*D*^, where *L* is the sequence length and *D* is the feature dimension. The query, key, and value matrices are computed as:


$$\begin{array}{l} Q=XW_{Q},\;K=XW_{K},\;V=XW_{V}, \end{array}$$ (1)

where $Q,K\in {\mathbb{R}}^{L\times d_{k}}$ and $V\in {\mathbb{R}}^{L\times d_{v}}$. The standard attention operation is:


$$\begin{array}{l} \text{Attention}\left(Q,K,V\right)=\text{Softmax}\left(\frac{QK^{T}}{\sqrt{d_{k}}}\right)V. \end{array}$$ (2)

Standard self-attention has quadratic complexity, *O*(*L*^2^), with respect to the sequence length. To reduce this cost, the key and value matrices are downsampled using a depthwise convolution with kernel size (*s*+1) × 1 and stride *s*, where *s* denotes the reduction stride. The reduced key and value matrices are denoted as *K*_*s*_ and *V*_*s*_. The efficient attention operation is then written as:


$$\begin{array}{l} \text{EA}\left(Q,K_{s},V_{s}\right)=\text{Softmax}\left(\frac{QK_{s}^{T}}{\sqrt{d_{k}}}\right)V_{s}. \end{array}$$ (3)

In the Efficient Transformer block, the input tensor *X* ∈ ℝ^*L*×*D*^ is projected into query, key, and value representations. For *h* attention heads, each head uses the projected dimensions *d*_*k*_ = *D*/*h* and *d*_*v*_ = *D*/*h*. To reduce the quadratic cost of standard self-attention, only the key and value sequences are reduced along the token dimension using a strided depthwise convolution. If the original token length is *L* and the reduction stride is *s*, the reduced key and value tensors have an approximate length of *L*_*s*_ = ⌈*L*/*s*⌉. The query tensor remains at length *L*, while *K*_*s*_ and *V*_*s*_ are used to compute reduced key-value attention. This reduces the attention complexity from *O*(*L*^2^) to approximately *O*(*LL*_*s*_), making the Transformer block more suitable for resource-constrained IoMT settings.

The output of the Efficient Transformer block is denoted as *X*′ ∈ ℝ^*L*×*D*^. SeqPool then performs attention-guided feature compression over the token dimension. A learnable projection vector $w_{p}\in {\mathbb{R}}^{D}$ first produces token-level attention scores:


$$\begin{array}{l} a=\mathrm{Softmax}\left(X^{\prime }w_{p}\right), \end{array}$$ (4)

where *a* ∈ ℝ^*L*^ represents the normalized importance weights of all tokens. The final compact representation is obtained through weighted pooling:


$$\begin{array}{l} z=\overset{L}{\underset{i=1}{\sum }}a_{i}X_{i}^{\prime }, \end{array}$$ (5)

where *z* ∈ ℝ^*D*^ is the compressed feature vector passed to the classifier. The final class probabilities are computed as:


$$\begin{array}{l} ŷ=\mathrm{Softmax}\left(W_{c}z+b_{c}\right), \end{array}$$ (6)

where *W*_*c*_ and *b*_*c*_ are the classifier weight matrix and bias term, respectively. This formulation clarifies that SeqPool is an attention-weighted pooling mechanism that converts the token-level Transformer output into a fixed-length representation for classification.

### 3.4 Federated learning framework

In the proposed FedLC-Trans framework, federated learning is used to train the intrusion detection model across distributed IoMT clients without transferring raw healthcare or network data to a central server As illustrated in Figure 2. Each client keeps its local dataset and trains a local copy of the FedLC-Trans model. Only model parameters are communicated to the central server for aggregation. This design reduces the privacy risks associated with centralized data collection while allowing the global model to learn from distributed IoMT environments.

#### 3.4.1 Local model training

To simulate realistic non-IID conditions in federated learning, the training data for each dataset were partitioned across K = 5 clients, with each client receiving a distinct subset of the data. The non-IID distribution was created using a Dirichlet distribution with α = 0.5, producing a moderate label skew across clients. Each client holds approximately the following number of samples: Client 1—22,000; Client 2—21,500; Client 3—23,000; Client 4—22,500; Client 5—21,800. Mini-batch sizes were kept consistent across clients (batch_size = 64). During each communication round, all clients participate in local training, and only model weight updates are transmitted to the central server for aggregation using the FedAvg Algorithm. To ensure reproducibility, the federated simulation was independently configured for each dataset. Each dataset was divided into five IoMT clients using a Dirichlet-based non-IID partition strategy (α = 0.5), which generates heterogeneous class distributions among clients. All clients maintained local training data throughout the federated process. Dataset-independent preprocessing steps, including normalization, Random Forest feature selection, and PCA projection, were learned exclusively from the global training partition before client distribution to guarantee identical feature spaces. In contrast, SMOTE-based oversampling was performed locally within each client training dataset to preserve realistic client-level imbalance characteristics.

The local objective function at client *k* is defined using cross-entropy loss:


$$\begin{array}{l} L_{k}\left(\theta_{k}^{t}\right)=-\frac{1}{N_{k}}\overset{N_{k}}{\underset{i=1}{\sum }}\overset{C}{\underset{c=1}{\sum }}y_{i,c}^{k}\log\left({ŷ}_{i,c}^{k}\right) \end{array}$$ (7)

where *C* is the number of classes, $y_{i,c}^{k}$ is the ground-truth label of sample *i* for class *c*, and ${ŷ}_{i,c}^{k}$ is the predicted probability produced by the local FedLC-Trans model. The local model parameters are updated by minimizing this loss on the client dataset:


$$\begin{array}{l} \theta_{k}^{t+1}=\theta_{k}^{t}-\eta \nabla L_{k}\left(\theta_{k}^{t}\right) \end{array}$$ (8)

where η denotes the learning rate and $\nabla L_{k}\left(\theta_{k}^{t}\right)$ represents the gradient of the local loss with respect to the model parameters. After local training and federated aggregation, the final global FedLC-Trans model is used for intrusion detection on the held-out testing set.

#### 3.4.2 Global model aggregation and inference

For evaluation, the trained global model is applied to the test set. Each sample passes through the MobileNet block for spatial feature extraction, the Efficient Transformer block for temporal dependencies, and SeqPool for feature compression. Class probabilities are computed via a softmax layer, and the predicted label is assigned as the class with the highest probability. The complete inference procedure is summarized in Algorithm 2.

Algorithm 2 FedLC-Trans Inference 
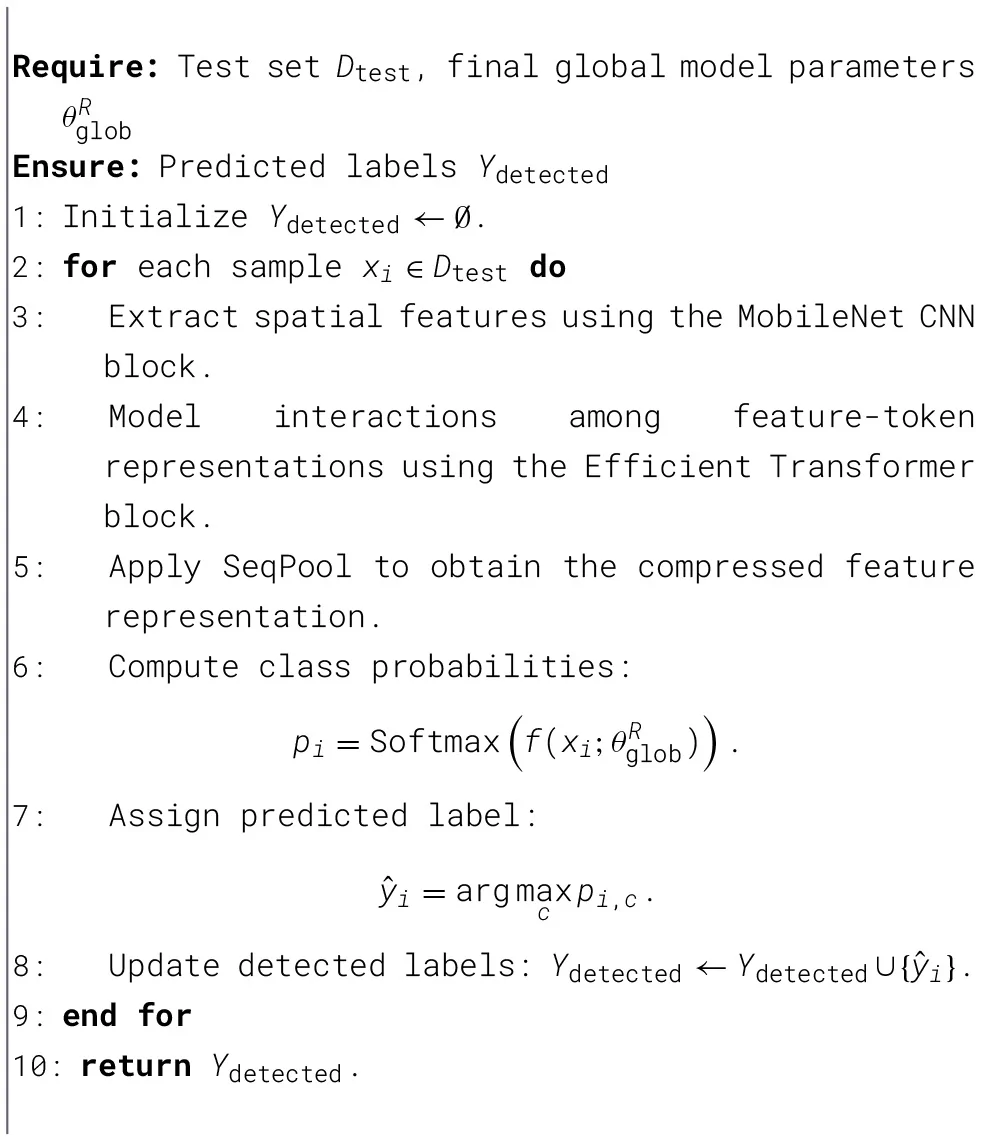

After local training, each client sends only its updated model parameters $\theta_{k}^{t+1}$ to the central server, while the raw IoMT data remain stored locally. The server aggregates the local updates using Federated Averaging (FedAvg). The global model for the next communication round is computed as:


$$\begin{array}{l} \theta_{\text{glob}}^{t+1}=\overset{K}{\underset{k=1}{\sum }}\frac{N_{k}}{N}\theta_{k}^{t+1}, \end{array}$$ (9)

where $N=\overset{K}{\underset{k=1}{\sum }}N_{k}$ is the total number of training samples across all clients. The updated global model $\theta_{\text{glob}}^{t+1}$ is redistributed to clients for the next training round. This process is repeated until convergence or until the maximum number of communication rounds is reached.

In each round, all five clients participate in local training (ρ = 1.0). Each client initializes its local model using the current global parameters, trains for five local epochs, and transmits updated model weights (not raw data) to the server. The server aggregates client models using sample-size-weighted FedAvg, ensuring that larger client partitions contribute proportionally to the global update while preserving a consistent model architecture and input feature space across all clients. Finally, after the global model has been obtained, inference is performed on the held-out testing set. Each test sample is processed by the trained LC-Trans model, and the resulting class predictions are generated according to the inference procedure summarized in Algorithm 2.

In the proposed FedLC-Trans framework, the central orchestration server has no access to raw IoMT traffic data, local feature matrices, client preprocessing outputs, or pooled training datasets during any stage of the federated lifecycle. Each client independently stores its local dataset and performs model training locally. The server only receives encrypted communication payloads containing model parameter updates and performs weighted FedAvg aggregation. Therefore, privacy preservation in this study refers specifically to preventing centralized raw-data collection. However, federated learning alone cannot guarantee complete privacy against advanced attacks such as gradient inversion, membership inference, or model reconstruction. Formal privacy mechanisms, including differential privacy and secure aggregation, are considered future extensions.

Algorithm 3 Local LC-Trans Training at Client *k* 
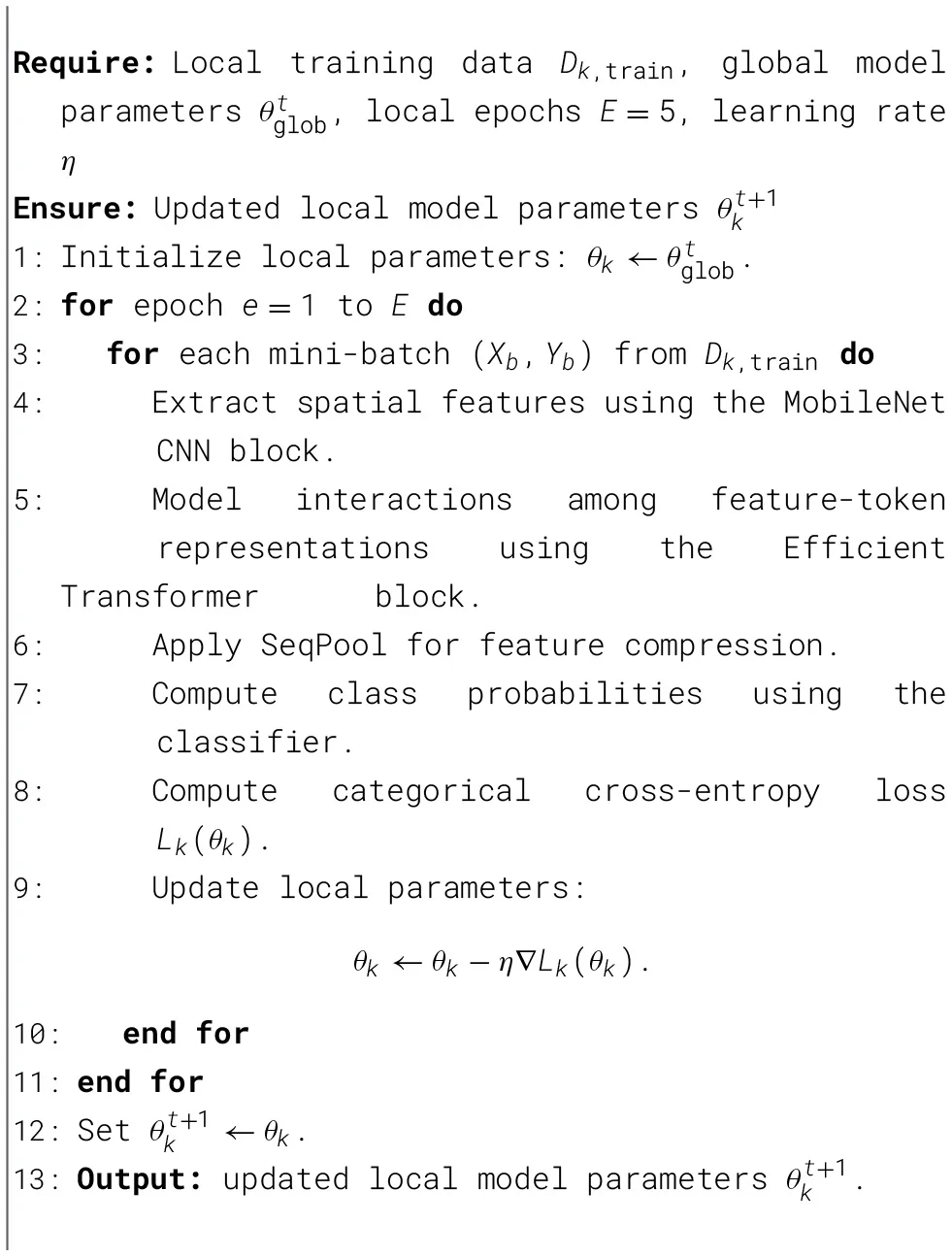

#### 3.4.3 Privacy and security considerations

In FedLC-Trans, raw IoMT traffic data remain locally stored and are not exchanged during federated training. Each client performs model optimization on its local data and transmits only model updates to the central server for aggregation. This decentralized training strategy reduces the need for centralized raw-data collection and thereby limits direct exposure of sensitive healthcare-related network information. However, federated learning alone does not provide a formal privacy guarantee and may still be vulnerable to advanced attacks such as gradient leakage, model inversion, and membership inference. Accordingly, the privacy claim in this study is restricted to reduced raw-data exposure through decentralized training. Differential privacy, secure aggregation, and additional cryptographic protection mechanisms are not implemented in the current framework and will be investigated in future work to strengthen privacy and security guarantees.

#### 3.4.4 Model convergence and evaluation

The federated training process continues over multiple communication rounds. Convergence is monitored using validation loss and classification metrics. After training, the final global model is evaluated using standard IDS metrics, including accuracy, precision, recall, F1-score, false positive rate, and misclassification rate.

This federated design enables collaborative intrusion detection while avoiding centralized collection of sensitive raw data. The lightweight MobileNet-Transformer architecture also supports computational efficiency, making the framework more practical for real-time IoMT networks.

To combine the local updates from all clients into a single global model, the FedAvg aggregation algorithm is applied at the server. Each client sends its locally updated model parameters to the server, which computes a weighted average based on the number of samples per client. The aggregated global model is then sent back to all clients for the next communication round. The complete aggregation procedure is summarized in Algorithm 4.

Algorithm 4 Federated Aggregation Using FedAvg 
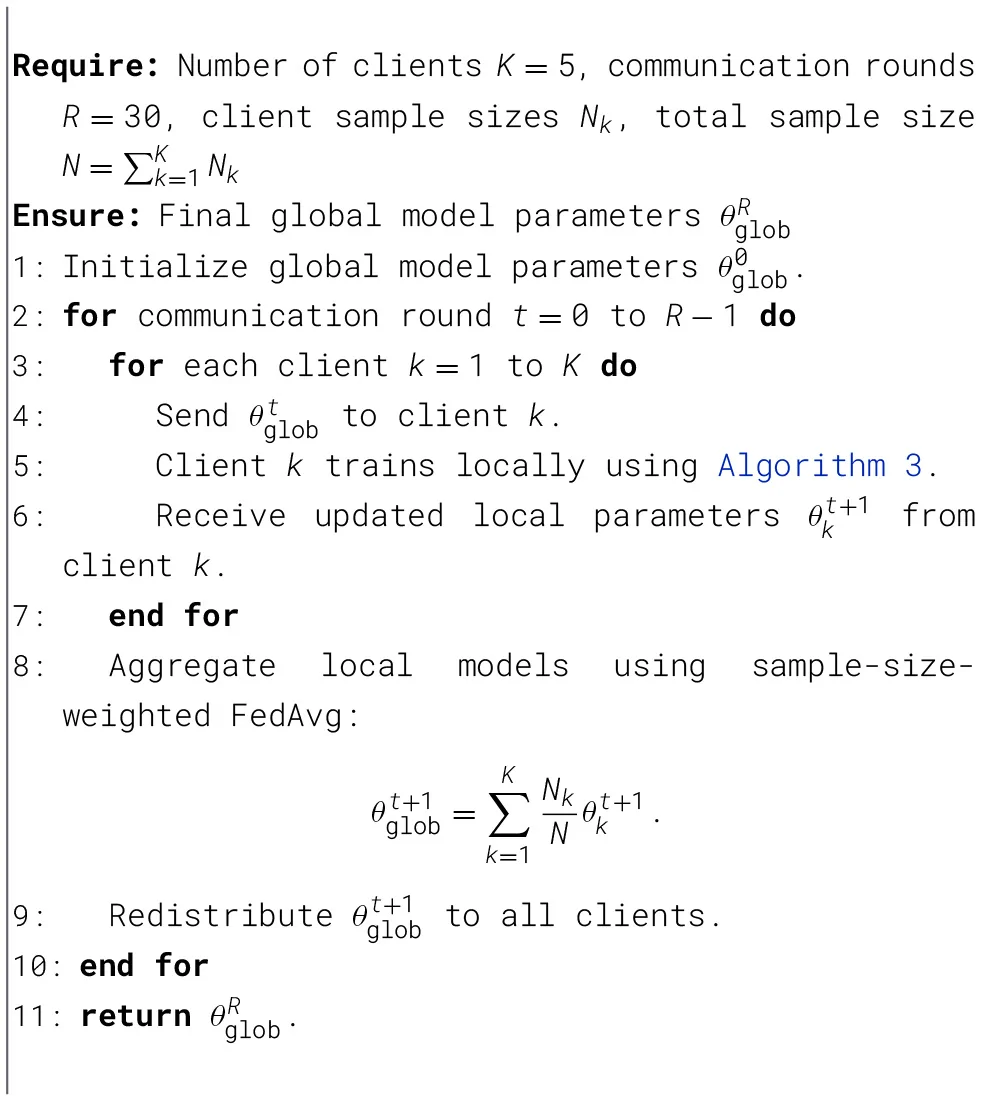

## 4 Experimental analysis

### 4.1 Datasets

This section provides an overview of three important datasets used in the development of IDS for the IoMT healthcare systems. WUSTL EHMS dataset (35), Table 2, ECU IoHT dataset (36) Table 3 dataset and CICIoMT2024 (37) dataset Table 4 are described.

**Table 2:** WUSTL-EHMS-2020 dataset distribution for proposed training and testing sets.

Class	Count	Category	Count	Attack types	Count	Proposed training (70%)	Proposed testing (30%)
Normal	14,272	Normal	14,272	Normal	14,272	9,990	4,282
Attack	2,046	Attack	2,046	Attack	2,046	1,432	614
Total	16,318		16,318		16,318	11,422	4,896

**Table 3:** ECU-IoHT dataset distribution for proposed training and testing sets.

Class	Count	Category	Count	Attack types	Count	Proposed training (70%)	Proposed testing (30%)
Benign	23,453	Benign	23,453	Normal	23,453	16,417	7,036
Attack	87,754	DoS	639	DoS attack	639	447	192
		Port scan	6,836	Nmap port scan	6,836	4,785	2,051
		Spoofing	2,359	ARP spoofing	2,359	1,651	708
		Smurf	77,920	Smurf attack	77,920	54,544	23,376
Total	111,207		111,207		111,207	77,844	33,363

**Table 4:** CICIoMT2024 dataset distribution for proposed training and testing sets.

Class	Count	Category	Count	Attack types	Count	Proposed training (70%)	Proposed testing (30%)
Benign	192,732	Benign	192,732	Benign	192,732	134,912	57,820
Attack	1,202,695	DoS	377,153	DDoS UDP flood 1	206,170	144,319	61,851
		DDoS	455,538	DDoS connect flood	173,036	121,125	51,911
		MQTT	262,938	DDoS SYN flood 1	144,258	100,981	43,277
		Recon	91,043	DoS UDP flood 1	137,011	95,908	41,103
		Spoofing	16,023	DoS SYN flood 1	111,412	77,988	33,424
				DDoS TCP flood 1	83,317	58,322	24,995
				Port scan	74,073	51,851	22,222
				DoS ICMP flood 1	73,635	51,545	22,090
				DoS TCP flood 1	55,095	38,567	16,528
				DoS publish flood	44,376	31,063	13,313
				DDoS publish flood	27,623	19,336	8,287
				DDoS ICMP flood 1	21,793	15,255	6,538
				DoS connect	12,773	8,941	3,832
				ARP spoofing	16,023	11,216	4,807
				OS scan	14,239	9,967	4,272
				Malformed data	5,130	3,591	1,539
				Recon VulScan	2,033	1,423	610
				Ping sweep	698	489	209
Total	1,395,427		1,395,427		1,395,427	976,799	418,628

### 4.2 Evaluation metrics

The performance of the proposed FedLC-Trans IDS is evaluated using several key metrics, including accuracy (Acc), precision (Pre), recall (Rec), F1-score (F1), false positive rate (FPR), misclassification rate (Misclass). These metrics are essential in assessing the model's effectiveness in detecting anomalies while maintaining a balance between false positives and false negatives, which is crucial in real-time IoMT applications.

Accuracy (Acc) measures the overall correctness of the model's predictions, defined as the ratio of correct predictions (both true positives and true negatives) to the total number of predictions:


$$\begin{array}{c} \text{Acc}=\frac{TP+TN}{TP+TN+FP+FN} \end{array}$$ (10)

Precision (Pre) focuses on the proportion of correctly predicted positive instances out of all instances predicted as positive, highlighting the model's ability to avoid false positives:


$$\begin{array}{c} \text{Pre}=\frac{TP}{TP+FP} \end{array}$$ (11)

Recall (Rec), also known as sensitivity, measures the proportion of actual positives that were correctly identified, which is important for detecting as many anomalies as possible:


$$\begin{array}{c} \text{Rec}=\frac{TP}{TP+FN} \end{array}$$ (12)

F1-score (F1) is the harmonic mean of Pre and Rec, providing a single metric that balances both the concerns of false positives and false negatives:


$$\begin{array}{c} \text{F1}=\frac{2\times TP}{2\times TP+FP+FN} \end{array}$$ (13)

The False Positive Rate (FPR) quantifies the rate of negative instances incorrectly classified as positive, helping assess how well the model avoids false alarms:


$$\begin{array}{c} \text{FPR}=\frac{FP}{FP+TN} \end{array}$$ (14)

The Misclassification Rate (Misclass) measures the overall proportion of incorrect predictions (false positives and false negatives) to the total number of instances:


$$\begin{array}{c} \text{Misclass}=\frac{FP+FN}{TP+TN+FP+FN} \end{array}$$ (15)

### 4.3 Implementation details

The LC-Trans backbone was designed to reduce parameter count, model size, and computational complexity. All experiments and computational profiling in this study were conducted using TensorFlow on an NVIDIA A100 GPU. On this platform, FedLC-Trans contained approximately 1.2 million parameters, required approximately 0.45 GFLOPs per forward pass, achieved an inference latency of approximately 12 ms per batch of 64 samples, occupied approximately 4.8 MB of storage, and produced a communication payload of approximately 0.8 MB per client during each federated-learning communication round.These measurements characterize the computational behavior of FedLC-Trans on the evaluated NVIDIA A100 platform. No deployment or hardware benchmarking was conducted on an actual embedded, mobile, or low-power IoMT edge device. Consequently, the reported inference latency should not be interpreted as evidence of equivalent real-time performance on resource-constrained IoMT hardware. In the present study, the term “lightweight” refers to the model's parameter count, model size, estimated FLOPs, and communication payload relative to the evaluated baseline architectures. Actual device-level inference latency, throughput, peak memory utilization, and energy consumption remain to be evaluated on representative embedded and low-power edge platforms.

The proposed FedLC-Trans architecture integrates MobileNet-based depthwise separable convolutional layers for spatial feature extraction, Efficient Transformer blocks for dependency modeling, and an attention-guided SeqPool module for feature compression. The models were trained using the Adam optimizer with a learning rate of 0.002, β_1_ = 0.9, β_2_ = 0.999, and ϵ = 10^−7^. A fixed mini-batch size of 64 was used consistently across all model configurations. For the federated-learning experiments, each dataset was divided into 70% training and 30% testing subsets. The transformed training data were distributed among five non-independent and identically distributed clients using a Dirichlet distribution with a concentration parameter of α = 0.5. All five clients participated in every communication round. Each client trained its local model for five epochs per round, and the resulting model updates were aggregated at the central server using sample-size-weighted Federated Averaging. The training process continued for 30 communication rounds. To prevent information leakage, normalization, Random Forest-based feature selection, and principal component analysis were fitted exclusively on the training partition. The normalization parameters, selected feature indices, and PCA projection matrix learned from the training data were subsequently applied to all client partitions and the held-out testing set. SMOTE was applied only to the local training data and was never applied to the testing partition. This procedure preserved a common feature space across clients while preventing testing information from influencing data balancing, feature selection, or dimensionality reduction.

To ensure a fair comparison, all baseline models were trained and evaluated using the same training–testing split, preprocessing pipeline, input feature space, optimizer settings, batch size, and evaluation metrics. For CNN-based baseline architectures originally developed for image data, the transformed IoMT feature vectors were reshaped into one-dimensional sequential feature maps rather than two-dimensional images. Consequently, MobileNet, EfficientNet, ShuffleNetV2, ResNet, Inception, and the other CNN-based models operated on the same feature representation as FedLC-Trans. The LSTM and Transformer baselines were also provided with the same ordered feature sequence so that the observed performance differences were primarily attributable to model architecture rather than differences in data preparation.

### 4.4 Exploratory cross-dataset statistical analysis

The primary experimental performance values were obtained from five repeated runs (*N* = 5). However, because the ablation results were summarized at the dataset level, an exploratory cross-dataset statistical analysis was conducted using the three benchmark datasets as matched blocks (*n* = 3). The MobileNet-only, Transformer-only, MobileNet–Transformer without federated learning, and complete FedLC-Trans configurations were treated as four related conditions within each dataset.For each performance metric, the Friedman test was applied to evaluate the overall differences among the four ablation configurations. Kendall's *W* was calculated as the corresponding omnibus effect-size measure. Pairwise comparisons between the complete FedLC-Trans framework and each ablated configuration were conducted using two-sided exact Wilcoxon signed-rank tests. Holm's procedure was applied to the three pairwise comparisons for each performance metric to control the family-wise error rate. Because the analysis was based on only three dataset-level aggregate observations, it was treated as exploratory cross-dataset evidence rather than confirmatory inference based on the five individual experimental runs. Statistical significance was defined at *p* < 0.05.

## 5 Experimental findings

This section presents the experimental evaluation of FedLC-Trans on three benchmark IoMT intrusion detection datasets: WUSTL-EHMS, ECU-IoHT, and CICIoMT. The proposed framework is compared with representative deep learning architectures, including a baseline CNN (38), MobileNet (29), ShuffleNetV2 (32), ResNet (33), Inception (39), and the standard Transformer (16). These models were selected to evaluate the relative contribution of lightweight convolutional feature extraction, sequential dependency modeling, and transformer-based attention mechanisms in IoMT intrusion detection.

To ensure a consistent comparison, all models were trained and evaluated using the same train-test split, preprocessing pipeline, input feature representation, and evaluation metrics. Model performance was assessed using six standard classification metrics: accuracy (Acc), precision (Pre), recall (Rec), F1-score (F1), false positive rate (FPR), and misclassification rate (Misclass). Together, these metrics provide a broader assessment of classification effectiveness, false-alarm behavior, and overall detection reliability across different IoMT datasets.

### 5.1 Robustness and validity considerations

The reported performance values are based on a held-out 30% test partition after applying a training-only preprocessing pipeline. To ensure reproducibility, all experiments were repeated N_runs = 5 times using different random seeds. The reported metrics (accuracy, precision, recall, F1-score, false positive rate, and misclassification rate) are presented as mean ± standard deviation over these runs. Confidence intervals were computed at 95% for key metrics to provide an estimate of variability.

Normalization, Random Forest-based feature selection, and PCA were fitted only on the training partition, and the testing data were transformed using only parameters learned from the training data. SMOTE was applied exclusively to the training data, and no synthetic samples were generated from the testing partition.

These procedures ensure a leakage-free preprocessing pipeline while providing transparent reporting of experimental variability.

After applying RF-based feature selection, all three IoMT datasets exhibited substantial improvements in classification performance. For example, the WUSTL-EHMS dataset accuracy increased from 0.8313 to 0.9087, while ECU-IoHT and CICIoMT improved from 0.8527 to 0.9149 and 0.8165 to 0.8833, respectively (Table 5). These results demonstrate that a systematic, leakage-free preprocessing pipeline–including normalization, SMOTE balancing, feature selection, and PCA significantly enhances the detection capability of IDS models in distributed IoMT networks.

**Table 5:** Performance of datasets before and after preprocessing.

Datasets	Acc
WUSTL-EHMS 2020 (Original dataset)	0.8313
WUSTL-EHMS 2020 (After features selection RF)	0.9087
ECU-IoHT (Original dataset)	0.8527
ECU-IoHT (After features selection RF)	0.9149
CICIoMT 2024 (Original dataset)	0.8165
CICIoMT 2024 (After features selection RF)	0.8833

### 5.2 Comparison of FedLC-Trans with baseline models on the IoMT datasets

Tables 6–8 summarize the classification performance of baseline models and FedLC-Trans across the WUSTL-EHMS, ECU-IoHT, and CICIoMT datasets, with all metrics reported as mean ± standard deviation (SD) over multiple runs. CNN provides basic spatial feature extraction but exhibits higher misclassification, while EfficientNet, ShuffleNetV2, ResNet variants, and Inception-v4 progressively improve feature representation and F1-scores. Transformer captures temporal dependencies, enhancing detection of sequential attack patterns.

**Table 6:** Classification performance of baseline models and FedLC-Trans on WUSTL-EHMS (Mean ± SD).

Methods	Acc	Pre	Rec	F1	FPR	Misclass
CNN	0.9317 ± 0.0021	0.9313 ± 0.0020	0.9316 ± 0.0022	0.9309 ± 0.0020	0.0583 ± 0.0025	0.0683 ± 0.0024
EfficientNet	0.9352 ± 0.0019	0.9353 ± 0.0018	0.9354 ± 0.0019	0.9352 ± 0.0019	0.0548 ± 0.0022	0.0648 ± 0.0021
ShuffleNetV2	0.9519 ± 0.0015	0.9511 ± 0.0016	0.9517 ± 0.0015	0.9507 ± 0.0015	0.0381 ± 0.0018	0.0481 ± 0.0017
ResNet-18	0.9587 ± 0.0012	0.9585 ± 0.0013	0.9587 ± 0.0012	0.9583 ± 0.0012	0.0313 ± 0.0015	0.0413 ± 0.0014
ResNet-50	0.9655 ± 0.0010	0.9652 ± 0.0011	0.9649 ± 0.0010	0.9643 ± 0.0011	0.0245 ± 0.0013	0.0345 ± 0.0012
Inception-v4	0.9723 ± 0.0009	0.9719 ± 0.0010	0.9722 ± 0.0010	0.9718 ± 0.0010	0.0223 ± 0.0012	0.0277 ± 0.0011
Transformer	0.9858 ± 0.0007	0.9858 ± 0.0007	0.9857 ± 0.0008	0.9856 ± 0.0007	0.0140 ± 0.0009	0.0142 ± 0.0008
FedLC-Trans	**0.9913** **±** **0.0005**	**0.9911** **±** **0.0005**	**0.9912** **±** **0.0005**	**0.9909** **±** **0.0005**	**0.0016** **±** **0.0003**	**0.0087** **±** **0.0004**

**Table 7:** Classification performance of baseline models and FedLC-Trans on the ECU-IoHT dataset (Mean ± SD).

Methods	Acc	Pre	Rec	F1	FPR	Misclass
CNN	0.8713 ± 0.0030	0.8713 ± 0.0030	0.8716 ± 0.0031	0.8712 ± 0.0030	0.1187 ± 0.0035	0.1287 ± 0.0034
EfficientNet	0.8966 ± 0.0025	0.8966 ± 0.0025	0.8967 ± 0.0026	0.8965 ± 0.0025	0.0934 ± 0.0030	0.1034 ± 0.0029
ShuffleNetV2	0.9093 ± 0.0022	0.9091 ± 0.0022	0.9093 ± 0.0022	0.9087 ± 0.0021	0.0807 ± 0.0025	0.0907 ± 0.0024
ResNet-18	0.9219 ± 0.0018	0.9244 ± 0.0019	0.9227 ± 0.0018	0.9208 ± 0.0018	0.0681 ± 0.0020	0.0781 ± 0.0019
ResNet-50	0.9345 ± 0.0015	0.9347 ± 0.0015	0.9340 ± 0.0015	0.9335 ± 0.0015	0.0555 ± 0.0018	0.0655 ± 0.0017
Inception-v4	0.9472 ± 0.0012	0.9469 ± 0.0012	0.9472 ± 0.0012	0.9461 ± 0.0012	0.0428 ± 0.0015	0.0528 ± 0.0014
Transformer	0.9725 ± 0.0008	0.9721 ± 0.0008	0.9728 ± 0.0008	0.9715 ± 0.0008	0.0205 ± 0.0010	0.0275 ± 0.0010
FedLC-Trans	**0.9892** **±** **0.0005**	**0.9893** **±** **0.0005**	**0.9894** **±** **0.0005**	**0.9893** **±** **0.0005**	**0.0016** **±** **0.0003**	**0.0108** **±** **0.0004**

**Table 8:** Classification performance of baseline models and FedLC-Trans on CICIoMT (Mean ± SD).

Methods	Acc	Pre	Rec	F1	FPR	Misclass
CNN	0.9017 ± 0.0025	0.9019 ± 0.0025	0.9016 ± 0.0025	0.9009 ± 0.0025	0.0883 ± 0.0030	0.0983 ± 0.0030
EfficientNet	0.9217 ± 0.0020	0.9213 ± 0.0020	0.9219 ± 0.0021	0.9215 ± 0.0020	0.0683 ± 0.0025	0.0783 ± 0.0025
ShuffleNetV2	0.9189 ± 0.0021	0.9191 ± 0.0021	0.9193 ± 0.0021	0.9181 ± 0.0020	0.0711 ± 0.0025	0.0811 ± 0.0024
ResNet-18	0.9406 ± 0.0015	0.9405 ± 0.0015	0.9405 ± 0.0015	0.9404 ± 0.0015	0.0494 ± 0.0020	0.0594 ± 0.0020
ResNet-50	0.9493 ± 0.0012	0.9492 ± 0.0012	0.9499 ± 0.0012	0.9487 ± 0.0012	0.0407 ± 0.0015	0.0507 ± 0.0015
Inception-v4	0.9577 ± 0.0010	0.9579 ± 0.0010	0.9575 ± 0.0010	0.9561 ± 0.0010	0.0323 ± 0.0012	0.0423 ± 0.0012
Transformer	0.9795 ± 0.0007	0.9792 ± 0.0007	0.9792 ± 0.0007	0.9791 ± 0.0007	0.0105 ± 0.0010	0.0205 ± 0.0010
FedLC-Trans	**0.9916** **±** **0.0005**	**0.9915** **±** **0.0005**	**0.9918** **±** **0.0005**	**0.9913** **±** **0.0005**	**0.0016** **±** **0.0003**	**0.0084** **±** **0.0004**

FedLC-Trans achieves the highest performance across all datasets by integrating MobileNet-based spatial extraction, Transformer based temporal modeling, and attention-guided feature compression within a federated learning framework. On WUSTL-EHMS, it achieves 0.9913 ± 0.0005 accuracy (0.9909 ± 0.0005 F1), improving 0.55 percentage points over Transformer and reducing misclassification to 0.0087 ± 0.0004. On ECU-IoHT, it reaches 0.9892 ± 0.0005 accuracy (0.9893 ± 0.0005 F1), a 1.67–2.67 percentage point improvement and misclassification 0.0108 ± 0.0004. On CICIoMT, FedLC-Trans achieves 0.9916 ± 0.0005 accuracy (0.9913 ± 0.0005 F1), improving 1.21 percentage points over Transformer and reducing misclassification to 0.0084 ± 0.0004. These results demonstrate that the combined spatial, temporal, and attention-guided modules with federated learning consistently enhance detection performance, stability, and generalization across heterogeneous IoMT traffic.

FedLC-Trans achieved an accuracy of 0.9916 ± 0.0005 and an F1-score of 0.9913 ± 0.0005, outperforming the Transformer baseline, which achieved an accuracy of 0.9795 ± 0.0007 and an F1-score of 0.9791 ± 0.0007. This corresponds to an accuracy improvement of 1.21 percentage points. FedLC-Trans also reduced the misclassification rate from 0.0205 ± 0.0010 to 0.0084 ± 0.0004, indicating a substantial reduction in overall prediction errors. These results suggest that the proposed architecture remains effective on larger and more diverse IoMT traffic data. The performance gains can be attributed to the complementary roles of the MobileNet CNN, Efficient Transformer, and SeqPool module, which jointly improve feature extraction, temporal modeling, and discriminative feature selection.

### 5.3 Multi-class performance evaluation of FedLC-Trans

Table 9 presents the class-level performance of FedLC-Trans on the ECU-IoHT and CICIoMT2024 datasets. To ensure mathematical consistency, the F1-score for each class was recalculated from the corresponding precision and recall values using *F*1 = 2*PR*/(*P*+*R*), while the misclassification rate was verified as 1 − Accuracy. On ECU-IoHT, the resulting F1-scores range from 0.9760 for Normal traffic to 0.9856 for ARP Spoofing, demonstrating consistently strong discrimination across both benign and malicious traffic categories. On CICIoMT2024, F1-scores range from 0.9448 for ARP Spoofing to 0.9866 for DDoS ICMP Flood, with strong performance observed across most DDoS, DoS, scanning, and spoofing classes. The variation among individual attack categories highlights the importance of examining per-class metrics in addition to aggregate performance measures. These results indicate that the combination of MobileNet-based local feature extraction, Transformer-based feature-token interaction modeling, and attention-guided SeqPool aggregation provides effective representations for multiclass IoMT intrusion detection. Nevertheless, the high performance observed for several classes should be interpreted with appropriate caution, considering potential dataset-specific characteristics and the adopted preprocessing and evaluation protocol.

**Table 9:** Experimental study of FedLC-Trans multi-class performance.

Class	Acc	Pre	Rec	F1	FPR	Misclass
ECU						
Normal	0.9872	0.9671	0.9850	0.9760	0.0134	0.0128
DoS attack	0.9762	0.9871	0.9791	0.9831	0.0240	0.0238
Nmap port scan	0.9749	0.9708	0.9887	0.9797	0.0258	0.0251
ARP spoofing	0.9838	0.9845	0.9868	0.9856	0.0165	0.0162
Smurf attack	0.9737	0.9786	0.9895	0.9840	0.0267	0.0263
CICIoMT						
Benign	0.9693	0.9775	0.9687	0.9731	0.0305	0.0307
DDoS UDP flood	0.9772	0.9839	0.9741	0.9790	0.0228	0.0228
DDoS connect flood	0.9738	0.9751	0.9640	0.9695	0.0262	0.0262
DDoS SYN flood	0.9852	0.9874	0.9847	0.9860	0.0148	0.0148
DoS UDP flood	0.9718	0.9743	0.9654	0.9698	0.0282	0.0282
DoS SYN flood	0.9691	0.9725	0.9709	0.9717	0.0309	0.0309
DDoS TCP flood	0.9734	0.9749	0.9686	0.9717	0.0266	0.0266
Port scan	0.9685	0.9698	0.9660	0.9679	0.0315	0.0315
DoS ICMP flood	0.9721	0.9739	0.9756	0.9747	0.0279	0.0279
DoS TCP flood	0.9765	0.9787	0.9727	0.9757	0.0235	0.0235
DoS publish flood	0.9763	0.9862	0.9770	0.9816	0.0237	0.0237
DDoS publish flood	0.9839	0.9762	0.9788	0.9775	0.0161	0.0161
DDoS ICMP flood	0.9877	0.9850	0.9882	0.9866	0.0123	0.0123
DoS connect	0.9863	0.9840	0.9677	0.9758	0.0137	0.0137
ARP spoofing	0.9497	0.9441	0.9456	0.9448	0.0503	0.0503
OS scan	0.9788	0.9694	0.9697	0.9695	0.0212	0.0212
Malformed data	0.9825	0.9729	0.9707	0.9718	0.0175	0.0175
Recon VulScan	0.9768	0.9615	0.9615	0.9615	0.0232	0.0232
Ping sweep	0.9634	0.9672	0.9578	0.9625	0.0366	0.0366

Figure 4 presents the training accuracy and loss curves across epochs for all compared models on the WUSTL-EHMS, ECU-IoHT, and CICIoMT datasets. Overall, all models exhibit stable learning with steadily increasing accuracy and exponentially decreasing loss, indicating effective optimization. The proposed FedLC-Trans consistently converges faster and achieves the highest accuracy with the lowest loss across all datasets, outperforming baseline models. In contrast, conventional CNN-based and lightweight architectures show slower convergence and lower final performance, reflecting limited representation capacity.

**Figure 4:**
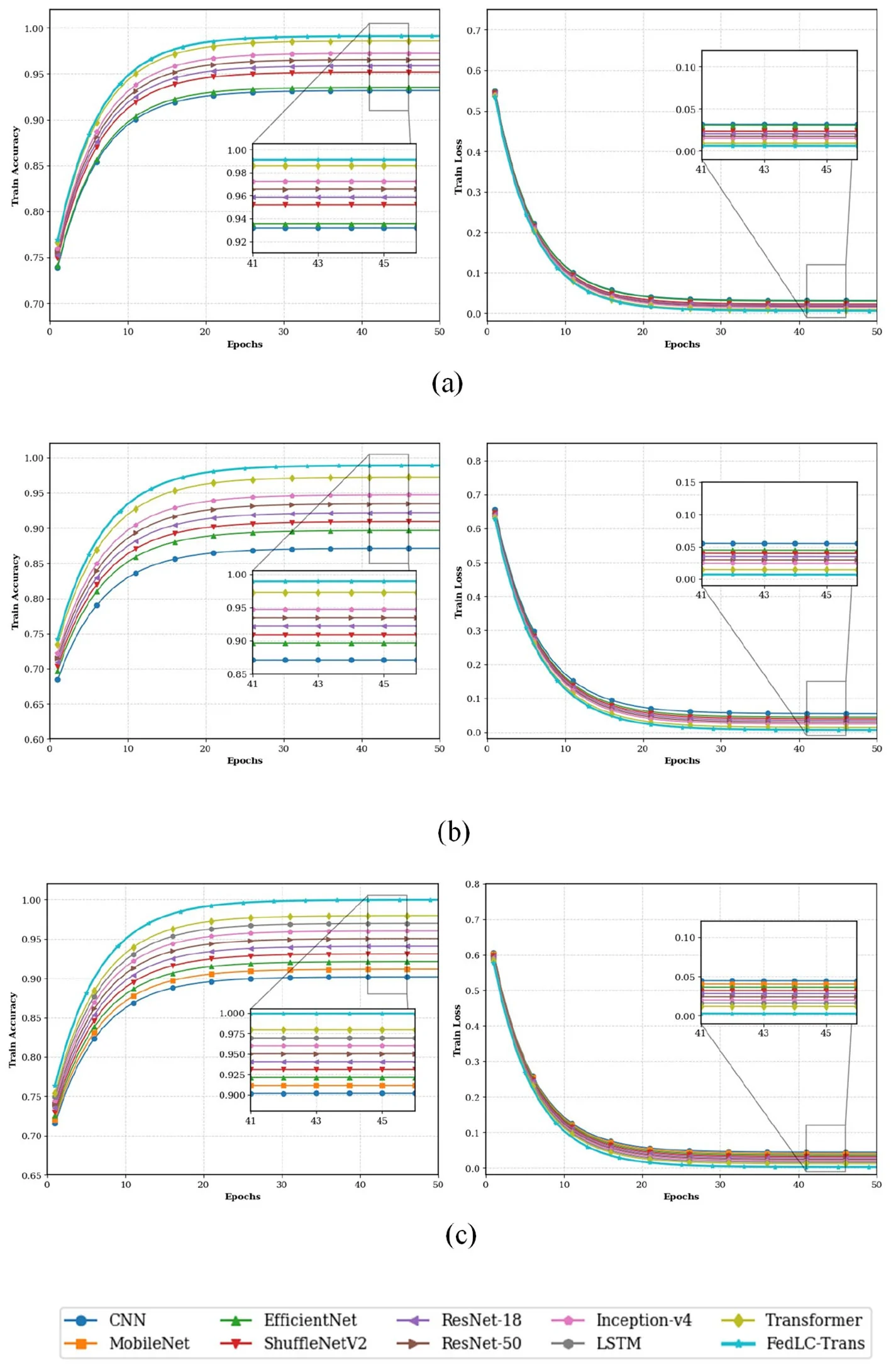
**(a)** Train accuracy and train loss on WUSTL-EHMS **(b)** Ecu-IoHT **(c)** CICIoMT datasets.

### 5.4 Ablation study

Table 10 presents an expanded ablation study evaluating the individual and combined contributions of the MobileNet feature extractor, Transformer module, SeqPool aggregation mechanism, and federated learning (FL). The results show that the contribution of each component varies across datasets, while their integration consistently provides the strongest overall performance. The MobileNet-only configuration achieved accuracies of 0.9631, 0.9631, and 0.9517 on WUSTL-EHMS, ECU-IoHT, and CICIoMT, respectively. The Transformer-only configuration obtained accuracies of 0.9693, 0.9592, and 0.9498, indicating that neither individual backbone consistently dominates across all datasets.

**Table 10:** Ablation study of FedLC-Trans components.

Dataset	MobileNet	Transformer	SeqPool	FL	Acc	Pre	Rec	F1
WUSTL-EHMS	✓	×	×	×	0.9631	0.9607	0.9621	0.9614
	×	✓	×	×	0.9693	0.9671	0.9678	0.9639
	✓	✓	✓	×	0.9858	0.9858	0.9857	0.9856
	✓	✓	×	✓	0.9891	0.9886	0.9885	0.9887
	✓	✓	✓	✓	0.9913	0.9911	0.9912	0.9909
ECU-IoHT	✓	×	×	×	0.9631	0.9607	0.9621	0.9614
	×	✓	×	×	0.9592	0.9562	0.9593	0.9621
	✓	✓	✓	×	0.9725	0.9721	0.9728	0.9715
	✓	✓	×	✓	0.9864	0.9865	0.9866	0.9865
	✓	✓	✓	✓	0.9892	0.9893	0.9894	0.9893
CICIoMT	✓	×	×	×	0.9517	0.9522	0.9518	0.9520
	×	✓	×	×	0.9498	0.9508	0.9491	0.9493
	✓	✓	✓	×	0.9795	0.9792	0.9792	0.9791
	✓	✓	×	✓	0.9889	0.9888	0.9890	0.9889
	✓	✓	✓	✓	0.9916	0.9915	0.9918	0.9913

When MobileNet, Transformer, and SeqPool were combined without federated learning, performance increased to 0.9858, 0.9725, and 0.9795 accuracy on WUSTL-EHMS, ECU-IoHT, and CICIoMT, respectively. This improvement indicates that the convolutional feature extractor, Transformer-based feature-interaction modeling, and attention-guided SeqPool aggregation provide complementary representation-learning capabilities even in the centralized configuration. To specifically evaluate the contribution of SeqPool under federated training, the full architecture was compared with an otherwise identical configuration in which SeqPool was removed. Without SeqPool, the federated model achieved accuracies of 0.9891, 0.9864, and 0.9889 on WUSTL-EHMS, ECU-IoHT, and CICIoMT, respectively, with corresponding F1-scores of 0.9887, 0.9865, and 0.9889. Incorporating SeqPool further increased the accuracies to 0.9913, 0.9892, and 0.9916 and the F1-scores to 0.9909, 0.9893, and 0.9913, respectively. These results indicate that attention-guided SeqPool provides an additional performance benefit by selectively aggregating informative feature-token representations before classification.

Overall, the full FedLC-Trans configuration, integrating MobileNet, Transformer, SeqPool, and federated learning, achieved the best performance across all three datasets. The ablation results therefore support the complementary roles of lightweight local feature extraction, Transformer-based feature-interaction modeling, attention-guided feature aggregation, and collaborative federated optimization in the proposed framework. The exploratory cross-dataset statistical analysis identified an overall difference among the four ablation configurations for accuracy, precision, recall, and F1-score (Table 11). For each performance metric, the Friedman test yielded $\chi_{\left(3\right)}^{2}=8.200$ and *p* = 0.0421, with Kendall's *W* = 0.911. The large Kendall's *W* value indicates a strong and consistent ordering of the four configurations across the three benchmark datasets. However, the exact Wilcoxon signed-rank comparisons between the complete FedLC-Trans framework and the individual ablated configurations were not statistically significant after Holm correction. Each raw pairwise *p*-value was 0.250, while the corresponding Holm-adjusted *p*-value was 0.750. Therefore, the results provide exploratory evidence of an overall configuration effect, but the individual pairwise improvements should be interpreted as consistent numerical gains rather than statistically confirmed superiority. The lack of statistically significant pairwise differences may be attributable to the limited number of matched datasets (*n* = 3), which reduces the statistical power of the *post-hoc* comparisons. Accordingly, these findings should be interpreted as exploratory cross-dataset evidence rather than confirmatory run-level statistical inference.

**Table 11:** Exploratory cross-dataset statistical analysis of the FedLC-Trans ablation configurations.

Metric	Matched datasets, *n*	Friedman $\chi_{\left(3\right)}^{2}$	*p*-value	Kendall's *W*	Full vs. MobileNet raw *p*	Full vs. Transformer raw *p*	Full vs. w/o FL raw *p*
Accuracy	3	8.200	0.0421	0.911	0.250	0.250	0.250
Precision	3	8.200	0.0421	0.911	0.250	0.250	0.250
Recall	3	8.200	0.0421	0.911	0.250	0.250	0.250
F1-score	3	8.200	0.0421	0.911	0.250	0.250	0.250

The three benchmark datasets were treated as matched blocks. The Friedman test used the conventional chi-square approximation. Pairwise comparisons were conducted using two-sided exact Wilcoxon signed-rank tests. Holm's procedure was applied separately to the three comparisons for each performance metric. The analysis is exploratory because it uses dataset-level aggregate results rather than the five individual experimental runs.

### 5.5 Comparison with previous IDS methods

Table 12 presents a cross-study comparison between FedLC-Trans and representative IDS methods reported in the literature. The comparison is included to position the proposed model relative to existing approaches on WUSTL-EHMS, ECU-IoHT, and CICIoMT datasets. However, because the reported studies may differ in preprocessing strategies, feature selection, class-balancing procedures, train-test splits, model configurations, and evaluation protocols, the results should be interpreted as an indicative benchmark rather than a strict one-to-one comparison.

**Table 12:** Comparison of existing methods with FedLC-Trans.

Dataset	Model	Acc	Pre	Rec	F1
WUSTL-EHMS	CNN-Focal (49)	0.9308	0.9423	0.7338	0.7963
	LSTM (50)	0.9991	0.9990	0.9991	0.9991
	MF-Transformer (21)	0.9988	0.9994	0.9990	0.9992
	RCLNet (22)	0.9978	0.9953	0.9983	0.9957
	FedLC-Trans	0.9913	0.9911	0.9912	**0.9909**
ECU-IoHT	Federated (51)	0.9759	0.9410	0.9074	0.9236
	KNN (50)	0.9770	0.8541	0.9421	0.9022
	BiLSTM (36)	0.9783	0.9835	0.9890	0.9862
	FedLC-Trans	0.9892	**0.9893**	0.9894	0.9893
CICIoMT	ML (37)	0.7350	0.7130	0.7650	0.6760
	SABiLSTM (52)	0.9656	0.9094	0.8379	0.8890
	SVM (53)	0.9318	0.9762	0.9264	0.9506
	FedLC-Trans	0.9916	0.9915	0.9918	0.9913

For the WUSTL-EHMS dataset, FedLC-Trans achieved high classification performance, with an accuracy of 0.9913 and an F1-score of 0.9909. Although some existing centralized models reported near-perfect results on this dataset, such differences may be influenced by dataset partitioning, preprocessing, and evaluation settings. In contrast, FedLC-Trans was evaluated under the proposed federated learning framework, where the model aims to preserve data decentralization while maintaining strong detection capability.

On the ECU-IoHT dataset, FedLC-Trans outperformed the compared methods across all reported metrics, achieving an accuracy of 0.9892, precision of 0.9893, recall of 0.9894, and F1-score of 0.9893. This improvement indicates that the proposed architecture can effectively learn discriminative attack patterns in IoHT traffic while maintaining a balanced trade-off between precision and recall.

Similarly, on the CICIoMT dataset, FedLC-Trans achieved the best performance among the listed methods, with an accuracy of 0.9916 and an F1-score of 0.9913. These results demonstrate the effectiveness of combining lightweight convolutional feature extraction, temporal dependency modeling, and federated optimization for intrusion detection in heterogeneous IoMT environments.

Overall, the comparative results suggest that FedLC-Trans provides robust and consistent detection performance across multiple healthcare-oriented IoT datasets. Nevertheless, some challenges remain, including computational overhead, limited interpretability.

## 6 Conclusion

This study proposed FedLC-Trans, a lightweight CNN–Transformer-based federated learning framework for intrusion detection in Internet of Medical Things (IoMT) networks. The architecture integrates MobileNet-based depthwise separable convolutions for efficient spatial feature extraction, an Efficient Transformer module for modeling dependencies in network traffic, and an attention-guided SeqPool module for compact feature representation. Federated learning enables collaborative model training across distributed IoMT clients while keeping raw traffic data locally stored.

Despite the reported results, several limitations remain. First, all training, inference, and computational profiling experiments were conducted on an NVIDIA A100 GPU, and FedLC-Trans was not deployed on an actual embedded or low-power IoMT device. Therefore, the reported inference latency of approximately 12 ms per batch of 64 samples cannot be generalized directly to resource-constrained medical sensors, gateways, mobile processors, or other edge platforms. Device-level performance may vary according to processor architecture, available memory, numerical precision, batch size, software optimization, and communication conditions. The present results demonstrate relative architectural efficiency but do not yet establish operational suitability for real-time embedded deployment. Second, although federated learning reduces centralized raw-data exposure, it does not provide formal protection against gradient leakage, model inversion, or membership-inference attacks. Third, the interpretability of the proposed model remains limited, which may affect its practical adoption in healthcare cybersecurity environments where transparent and auditable decisions are required.

Future work will evaluate FedLC-Trans on representative embedded and low-power edge platforms and report device-level inference latency, throughput, peak memory utilization, energy consumption, and federated communication cost. Model quantization, pruning, knowledge distillation, and hardware-aware optimization will also be investigated to improve practical deployment efficiency. In addition, secure aggregation, differential privacy, communication-efficient federated optimization, and lightweight explainable artificial intelligence methods will be explored to strengthen the privacy, efficiency, interpretability, and practical trustworthiness of the proposed framework.

## Data Availability

The data is available on request from the corresponding author.
